# Supplementary material for: HIVprotI: an integrated web based platform for prediction and design of HIV proteins inhibitors
Source: J Cheminform. 2018 Mar 9;10:12. doi: 10.1186/s13321-018-0266-y (PMC5845081; doi:10.1186/s13321-018-0266-y)
Supplement: Supplementary file 2 — Additional file 2. Source code of HIVProtI web server. [file 13321_2018_266_MOESM2_ESM.zip › HIVprotI_Source-code/hivprotI/weka-3-6-6/doc/weka/core/converters/package-tree.html]

weka.core.converters Class Hierarchy


---


|  |  |  |
| --- | --- | --- |
| **Overview** \| **Package** \| Class \| **Tree** \| **Deprecated** \| **Index** \| **Help** \| **Weka's home** | |  |
| **PREV**   **NEXT** | **FRAMES**    **NO FRAMES**     **All Classes** |  |


---


## Hierarchy For Package weka.core.converters

**Package Hierarchies:**: All Packages

---

## Class Hierarchy

- java.lang.Object
  - weka.core.converters.**AbstractLoader** (implements weka.core.converters.Loader)
    - weka.core.converters.**AbstractFileLoader** (implements weka.core.EnvironmentHandler, weka.core.converters.FileSourcedConverter)
      - weka.core.converters.**ArffLoader** (implements weka.core.converters.BatchConverter, weka.core.converters.IncrementalConverter, weka.core.converters.URLSourcedLoader)- weka.core.converters.**C45Loader** (implements weka.core.converters.BatchConverter, weka.core.converters.IncrementalConverter)- weka.core.converters.**CSVLoader** (implements weka.core.converters.BatchConverter, weka.core.OptionHandler)- weka.core.converters.**LibSVMLoader** (implements weka.core.converters.BatchConverter, weka.core.converters.URLSourcedLoader)- weka.core.converters.**SerializedInstancesLoader** (implements weka.core.converters.BatchConverter, weka.core.converters.IncrementalConverter)- weka.core.converters.**SVMLightLoader** (implements weka.core.converters.BatchConverter, weka.core.converters.URLSourcedLoader)- weka.core.converters.**XRFFLoader** (implements weka.core.converters.BatchConverter, weka.core.converters.URLSourcedLoader)
      - weka.core.converters.**DatabaseLoader** (implements weka.core.converters.BatchConverter, weka.core.converters.DatabaseConverter, weka.core.converters.IncrementalConverter, weka.core.OptionHandler)- weka.core.converters.**TextDirectoryLoader** (implements weka.core.converters.BatchConverter, weka.core.OptionHandler)
    - weka.core.converters.**AbstractSaver** (implements weka.core.CapabilitiesHandler, weka.core.converters.Saver)
      - weka.core.converters.**AbstractFileSaver** (implements weka.core.EnvironmentHandler, weka.core.converters.FileSourcedConverter, weka.core.OptionHandler)
        - weka.core.converters.**ArffSaver** (implements weka.core.converters.BatchConverter, weka.core.converters.IncrementalConverter)- weka.core.converters.**C45Saver** (implements weka.core.converters.BatchConverter, weka.core.converters.IncrementalConverter, weka.core.OptionHandler)- weka.core.converters.**CSVSaver** (implements weka.core.converters.BatchConverter, weka.core.converters.FileSourcedConverter, weka.core.converters.IncrementalConverter)- weka.core.converters.**LibSVMSaver** (implements weka.core.converters.BatchConverter, weka.core.converters.IncrementalConverter)- weka.core.converters.**SerializedInstancesSaver** (implements weka.core.converters.BatchConverter)- weka.core.converters.**SVMLightSaver** (implements weka.core.converters.BatchConverter, weka.core.converters.IncrementalConverter)- weka.core.converters.**XRFFSaver** (implements weka.core.converters.BatchConverter)
        - weka.core.converters.**DatabaseSaver** (implements weka.core.converters.BatchConverter, weka.core.converters.DatabaseConverter, weka.core.converters.IncrementalConverter, weka.core.OptionHandler)
      - weka.core.converters.**ArffLoader.ArffReader** (implements weka.core.RevisionHandler)- weka.core.converters.**ConverterUtils** (implements weka.core.RevisionHandler, java.io.Serializable)- weka.core.converters.**ConverterUtils.DataSink** (implements weka.core.RevisionHandler, java.io.Serializable)- weka.core.converters.**ConverterUtils.DataSource** (implements weka.core.RevisionHandler, java.io.Serializable)- weka.experiment.**DatabaseUtils** (implements weka.core.RevisionHandler, java.io.Serializable)
                - weka.core.converters.**DatabaseConnection**

## Interface Hierarchy

- weka.core.converters.**BatchConverter**- weka.core.converters.**DatabaseConverter**- weka.core.converters.**FileSourcedConverter**- weka.core.converters.**IncrementalConverter**- weka.core.**RevisionHandler**
          - weka.core.converters.**Loader** (also extends java.io.Serializable)- weka.core.converters.**Saver** (also extends java.io.Serializable)
          - java.io.Serializable
            - weka.core.converters.**Loader** (also extends weka.core.RevisionHandler)- weka.core.converters.**Saver** (also extends weka.core.RevisionHandler)
            - weka.core.converters.**URLSourcedLoader**

---


|  |  |  |
| --- | --- | --- |
| **Overview** \| **Package** \| Class \| **Tree** \| **Deprecated** \| **Index** \| **Help** \| **Weka's home** | |  |
| **PREV**   **NEXT** | **FRAMES**    **NO FRAMES**     **All Classes** |  |


---
